# Supplementary material for: Structure-function analysis of purified proanthocyanidins reveals a role for polymer size in suppressing inflammatory responses
Source: Commun Biol. 2021 Jul 21;4:896. doi: 10.1038/s42003-021-02408-3 (PMC8295316; doi:10.1038/s42003-021-02408-3)
Supplement: Supplementary file 2 — Supplementary Information [file 42003_2021_2408_MOESM2_ESM.pdf]

Supplementary Information

A)

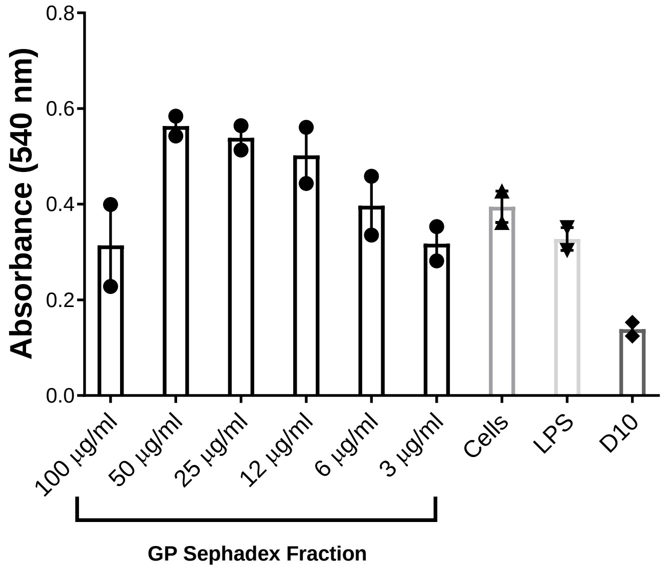

B)

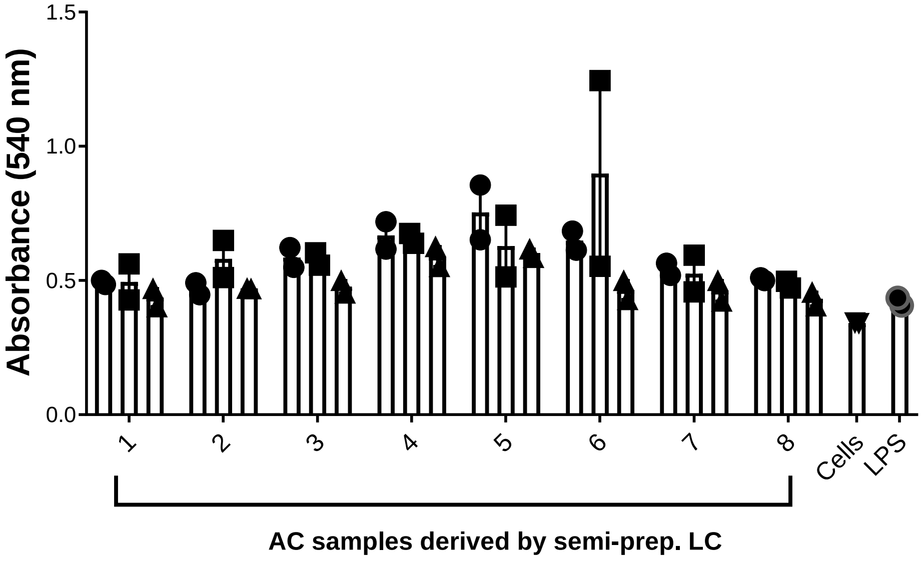

Supplementary Figure 1 Cytotoxicity test by Neutral Red Assay

A) Sephadex fraction were tested in LPS-activated RAW 264.7 macrophages at different concentrations (3-100 µg/ml) in duplicates. No signs of toxicity were reported.

B) Samples derived by semi-preparative liquid chromatography were tested in LPS-activated RAW 264.7 macrophages at a concentration of 15 µg/ml in duplicates. No signs of toxicity were reported as no absorbance values were below the control samples.

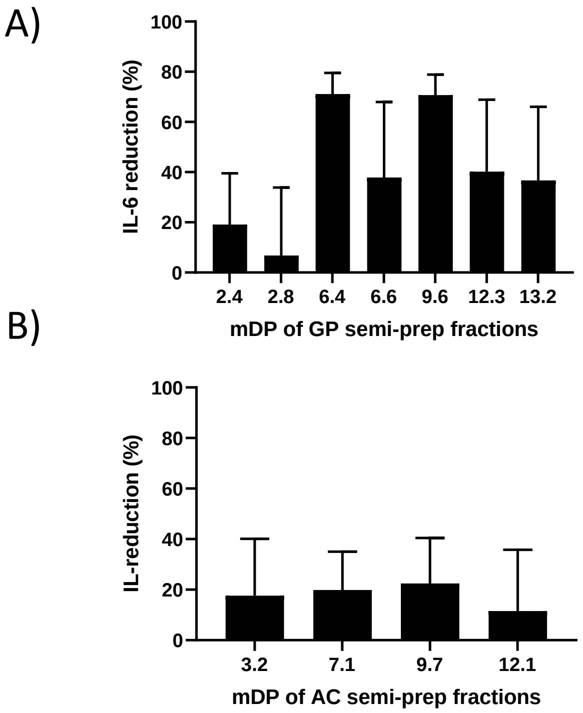

Supplementary Figure 2 - Inhibition of IL-6 secretion in LPS-activated macrophages stimulated with grape pomace or alpine currant PAC at equimolarity of 7.8 µM. Experiments were conducted at least twice with triplicate samples.

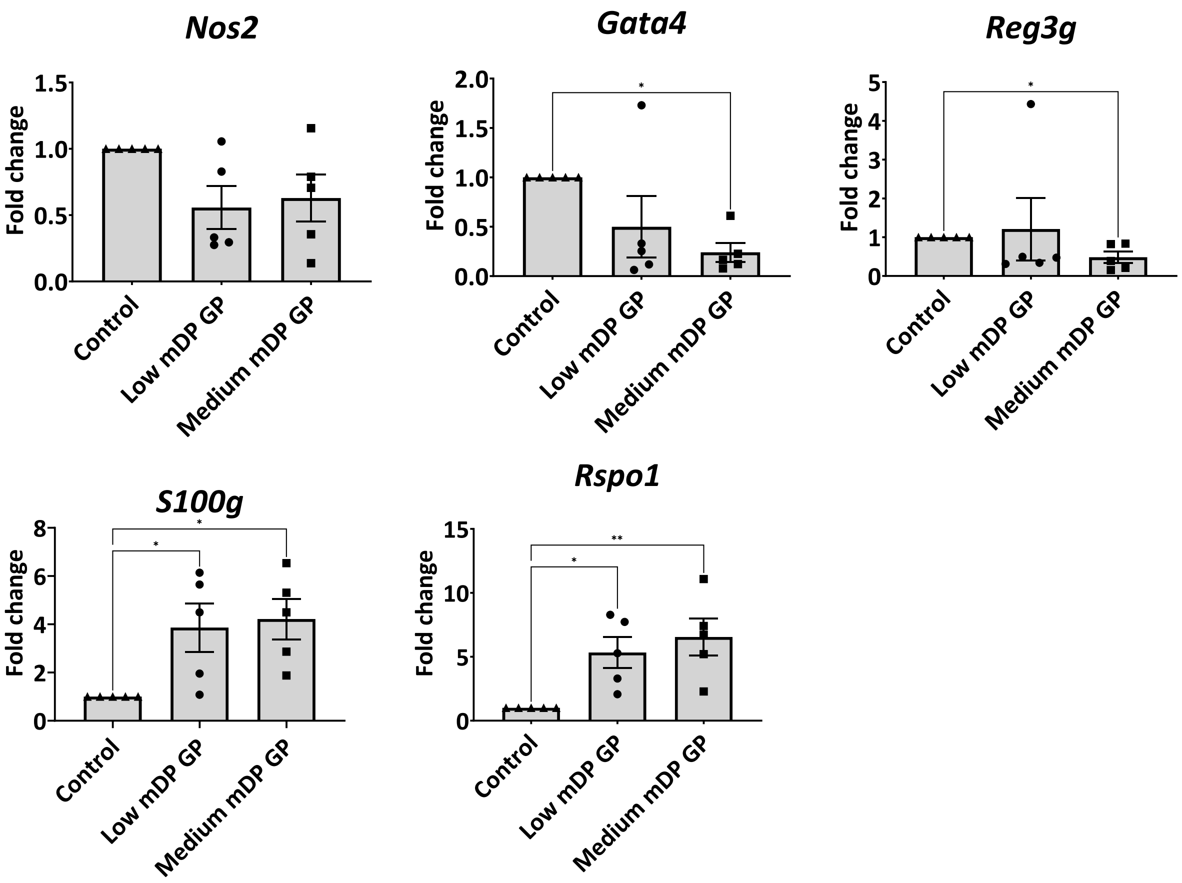

Supplementary Figure 3 - Regulation of gene expression in mouse ileum tissue by PAC

qPCR data depicting the regulation of *Nos2*, *Gata4*, *reg3g*, *S100g* and *Rspo1* in the ileum tissue of mice dosed with either low or medium mDP Sephadex fractions derived from grape pomace (GP). Data is expressed as fold changes relative to mice dosed with water only (*n*= 5 mice per treatment group). (***p* < 0.05, ****p* < 0.01 by Kruskal-Wallis test).**

A)

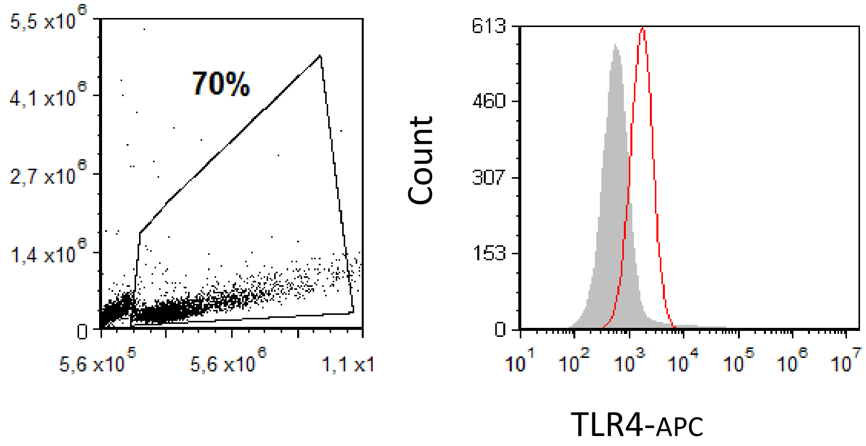

B)

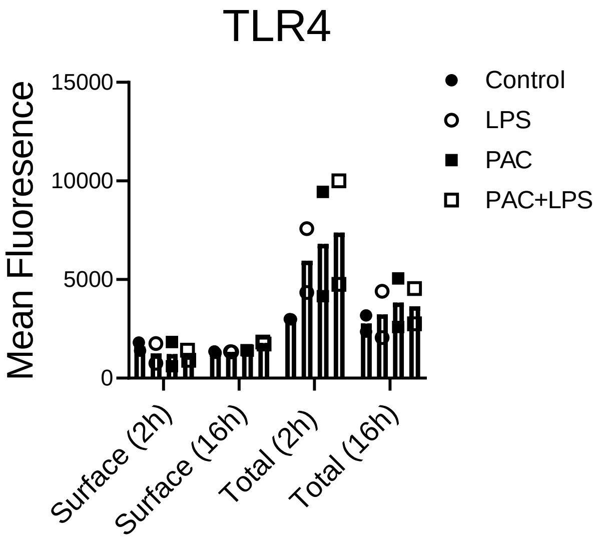

Supplementary Figure 4 Proanthocyanidins do not reduce TLR4 protein expression

A) Macrophages were gated on FFS/SSC and the TLR4 expression fluorescence intensity assessed. Shown is representative s catter plot and histogram (Surface TLR4 expression in cells compared to isotype control (shaded histogram) **B)** No effect of proanthocyanidin was observed on either surface or total expression of TLR4 proteins in LPS-activates RAW 264.7 macrophages stimulated with proanthocyanidins compared to appropriate controls.

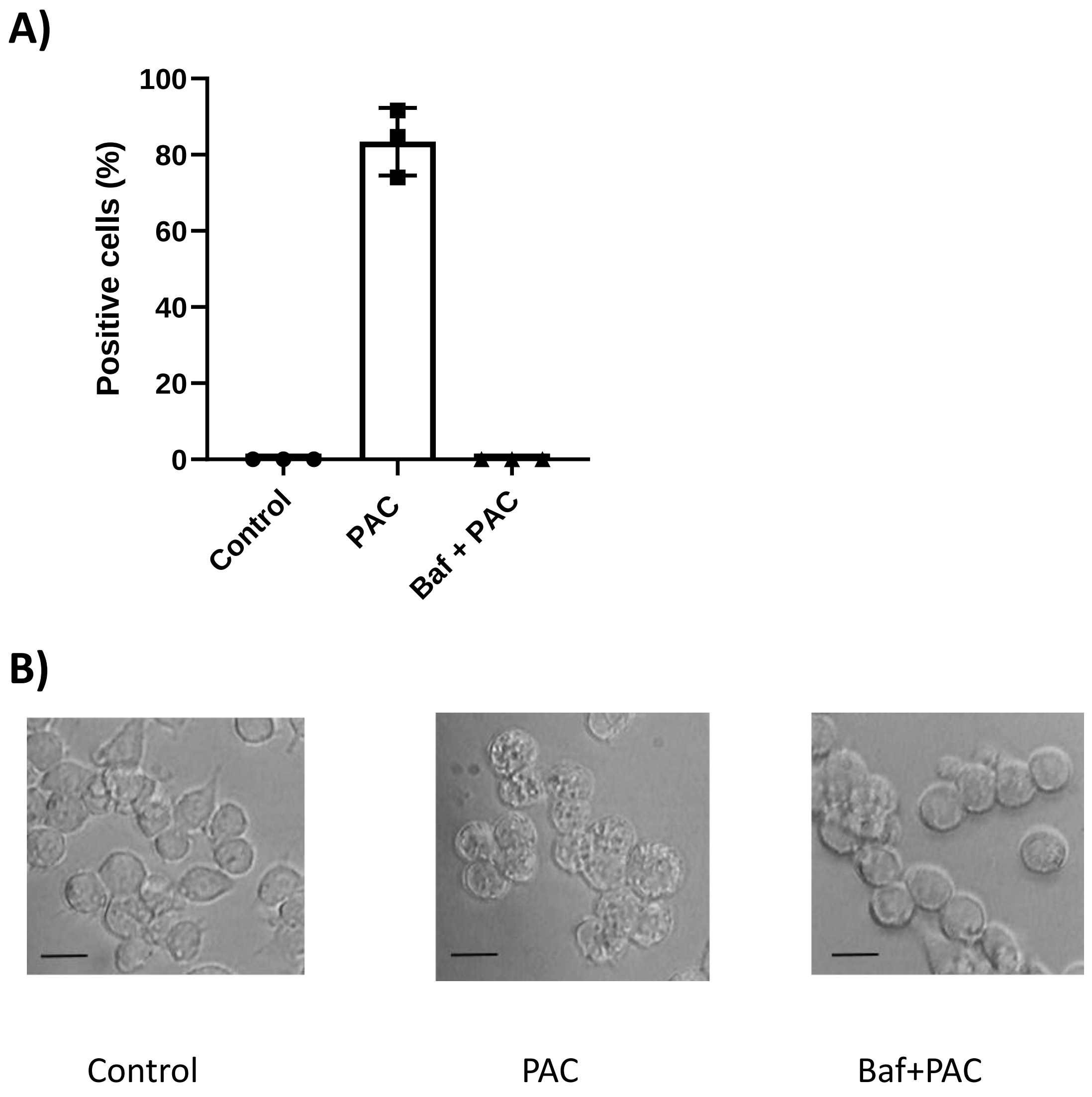

Supplementary Figure 5 Proanthocyanidins induce autophagosome-like structures in RAW 264.7 macrophages

A) RAW 264.7 macrophages stimulated with PAC (15 µg/ml) containing autophagosomes-like structures (= positive cells) were enumerated. Co-stimulation with Bafilo mycin (10nM) and unstimulated cells did not display autophagosomes-like structures. ND = Not detected. B) Microscopy pictures of RAW 264.7 cells with appropriate treatments. Scale bars indicate 10 µm.

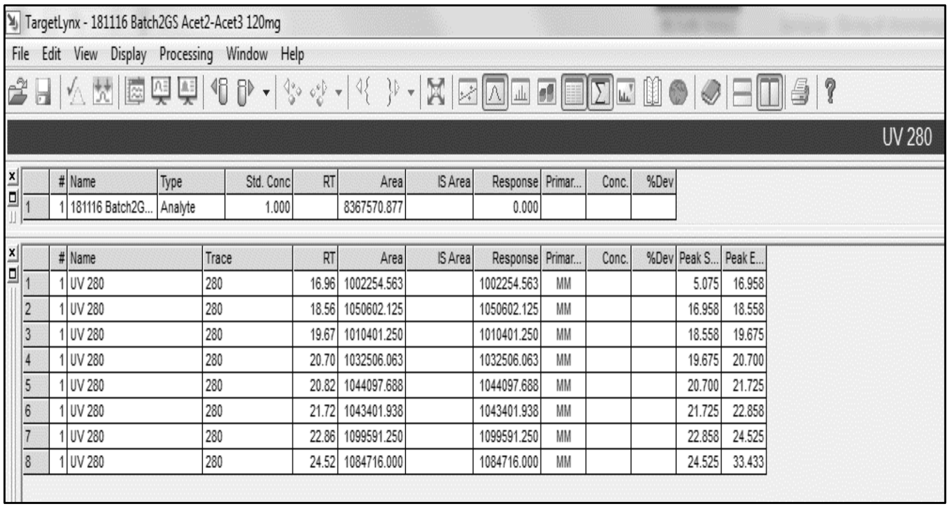

TargetLynx - 181116 Batch2GS Acet2-Acet3 120mg

File Edit View Display Processing Window Help

UV 280

	#	Name	Type	Std. Conc	RT	Area	IS Area	Response	Primar...	Conc.	%Dev
1	1	181116 Batch2G...	Analyte	1.000		8367570.877		0.000			

	#	Name	Trace	RT	Area	IS Area	Response	Primar...	Conc.	%Dev	Peak S...	Peak E...
1	1	UV 280	280	16.96	1002254.563		1002254.563	MM			5.075	16.958
2	1	UV 280	280	18.56	1050602.125		1050602.125	MM			16.958	18.558
3	1	UV 280	280	19.67	1010401.250		1010401.250	MM			18.558	19.675
4	1	UV 280	280	20.70	1032506.063		1032506.063	MM			19.675	20.700
5	1	UV 280	280	20.82	1044097.688		1044097.688	MM			20.700	21.725
6	1	UV 280	280	21.72	1043401.938		1043401.938	MM			21.725	22.858
7	1	UV 280	280	22.86	1099591.250		1099591.250	MM			22.858	24.525
8	1	UV 280	280	24.52	1084716.000		1084716.000	MM			24.525	33.433

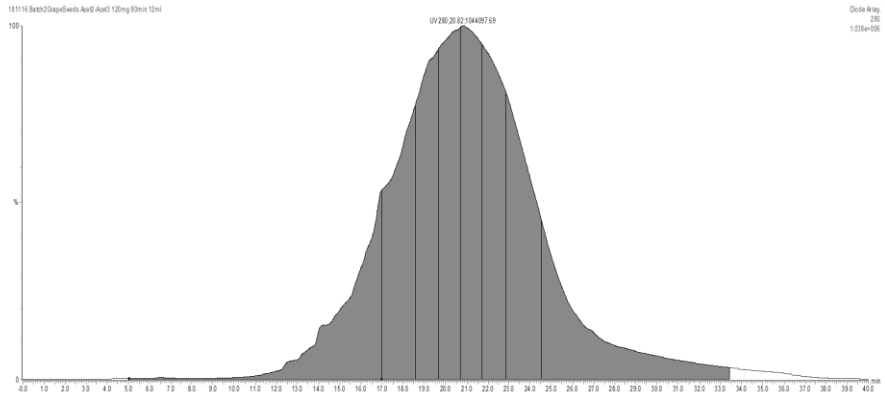

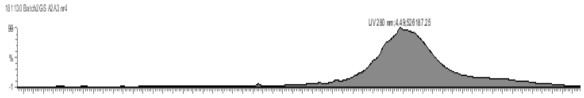

Supplementary Figure 6 - Integration of Chromatograms

MassLynx was used in order to determine the Area under the curve (AUC) within the time-span of 5–33 minutes. The Peak Response Area value was divided into 8 equally sized “slices”, corresponding to the 8 areas of the chromatogram. The 168 tubes resulting from the semi-preparative liquid-chromatography were then pooled accordingly into 8 highly purified PAC samples, which were individually analyzed by UPLC-MS/MS.

Supplementary Table 1 - List of material used for the extraction and purification of proanthocyanidins

	Material	References
Samples	Alpine currant (AC) Macerated in 80 % analytical acetone (09-08-2018)	This study
	Grape pomace (GP) Macerated in 80 % analytical acetone (14-09-2018)	Nor-Feed A/S (Hvidovre, Denmark)
Solvents	Acetyl acetate	VWR International S.A.S, France
	Analytical acetone	VWR International S.A.S, France
	Butanol	VWR International S.A.S, France
	Diethyl ether	VWR International S.A.S, France
	Ethyl acetate	VWR International S.A.S, France
	Formic acid 99-100%	WR International, EC
	Formic acid, LC-MS grade	Sigma Aldrich, USA
	Methanol, analytical grade	VWR International S.A.S, France
	Acetonitrile, LC-MS grade	VWR International S.A.S. (USA)
Other	MilliQ water Purified with Millipore Synergy UV system	Merck KGaA, Darmstadt, Germany
	Sephadex LH-20	GE Healthcare

Supplementary Table 2 - Extraction and purification of proanthocyanidins

Overview of the extraction and purification steps used to isolate purified PAC from grape pomace (GP) and alpine currant (AC) by Sephadex LH-20 fractionation followed by semi-preparative liquid chromatography.

<table>
<tr><th></th><th>Alpine currant</th><th>Grape pomace</th></tr>
<tr><td rowspan="7">1. Extraction by filtration</td><td>Macerated in 80 % analytical acetone (09-08-2018)</td><td>Macerated in 80 % analytical acetone (14-08-2018)</td></tr>
<tr><td colspan="2">5 extractions through Büchner funnel</td></tr>
<tr><td colspan="2">Evaporation of acetone from samples in fume hood</td></tr>
<tr><td colspan="2">UPLC analysis</td></tr>
<tr><td>Pooling of extractions 1-5</td><td>Pooling of extractions 1-5</td></tr>
<tr><td>Liquid-liquid extraction with ethyl acetate and butanol</td><td>-</td></tr>
<tr><td>-</td><td>-</td></tr>
<tr><td rowspan="4">2. Sephadex fractionation</td><td>Sephadex fractionation
6 water fractions
5 methanol fractions
6 acetone fraction</td><td>Sephadex fractionation
6 water fractions
5 methanol fractions
6 acetone fraction</td></tr>
<tr><td colspan="2">O/N evaporation of acetone fractions
Rotary evaporation of methanol fractions</td></tr>
<tr><td colspan="2">Freeze-drying and weighing of samples</td></tr>
<tr><td>Pooling of samples based on similarity of chromatogram → 6 alpine currant sephadex samples</td><td>Pooling of samples based on similarity of chromatogram → 8 grape pomace sephadex samples</td></tr>
<tr><td rowspan="5">3. Semi-preparative liquid chromatography</td><td colspan="2">Injection of ~120 mg of each sephadex fractions</td></tr>
<tr><td colspan="2">Collection of samples in 168 Eppendorf tubes</td></tr>
<tr><td>Pooling of each semi-prep run into 8 equal sub-fractions → 48 alpine currant semi-prep fractions</td><td>Pooling of each semi-prep run into 8 equal sub-fractions → 64 grape pomace semi-prep fractions</td></tr>
<tr><td colspan="2">Evaporation of solvents by rotavap, rotary evaporation or centrifugal concentration
and freeze-drying</td></tr>
<tr><td colspan="2">A total of 112 semi-prep samples containing highly purified PAC were generated with sample
weights ranging between 2-17 mg</td></tr>
</table>

Supplementary Table 3 - Primer sequences used in experiments

Primer name	Primer sequence (5'-> 3')
**Used for *in-vitro* studies**	
Tlr4 forward primer	*ACTGGCCTTTCAGGAACTTT*
Tlr4 reverse primer	*ACATCCTAGGGCTGTCTTTCTT*
Atp6v0d2 forward primer	*GGGCCAGTGTTCAGTTGCTA*
Atp6v0d2 reverse primer	*TCCTGCTGAGTTAGGAGGCT*
Rab7b forward primer	*GGAAGTGGCCTCTCACCAAA*
Rab7b reverse primer	*CCTCACACAGGTGGGAGTTC*
Gapdh forward primer	*TATGTCGTGGAGTCTACTGGT*
Gapdh reverse primer	*GAGTTGTCATATTTCTCGTGG*
**Used for *in-vivo* studies**	
Nos2 forward primer	*GGTGAAGGGACTGAGCTGTT*
Nos2 reverse primer	*TGCACTTCTGCTCCAAATCCA*
Gata4 forward primer	*TTCTGGGAAACTGGAGCTGG*
Gata4 reverse primer	*TGCTTTCTGCCTGCTACACA*
Reg3g forward primer	*CACCATCCTAGGGATCTGCAA*
Reg3g reverse primer	*ATGGGGCATCTTTCTTGGCA*
S100g forward primer	*GGAGCTGGATAAGAATGGCGA*
S100g reverse primer	*AGAGCGTGCGTTCAATCAGT*
Rspo1 forward primer	*TGTACTTACACAAGGGCCGC*
Rspo1 reverser primer	*GGGACCACTCGCTCATTTCA*
Gapdh forward primer	*TATGTCGTGGAGTCTACTGGT*
Gapdh reverse primer	*GAGTTGTCATATTTCTCGTGG*
