# Supplementary material for: Structure-function analysis of purified proanthocyanidins reveals a role for polymer size in suppressing inflammatory responses
Source: Commun Biol. 2021 Jul 21;4:896. doi: 10.1038/s42003-021-02408-3 (PMC8295316; doi:10.1038/s42003-021-02408-3)
Supplement: Supplementary file 3 — Description of Additional Supplementary Files [file 42003_2021_2408_MOESM3_ESM.pdf]

Description of Additional Supplementary Files

File Name: Supplementary Data 1
Description: Regulated gene pathways in RAW264.7 macrophages identified by gene-set enrichment analysis.

File Name: Supplementary Data 2
Description: Down-regulated gene pathways in PACtreated mice identified by gene-set enrichment analysis.

File Name: Supplementary Data 3
Description: Raw data underlying figures.

File Name: Supplementary Data 4
Description: Raw image files.
