# Supplementary figures and images for: Structure-function analysis of purified proanthocyanidins reveals a role for polymer size in suppressing inflammatory responses
Source: Commun Biol. 2021 Jul 21;4:896. doi: 10.1038/s42003-021-02408-3 (PMC8295316; doi:10.1038/s42003-021-02408-3)

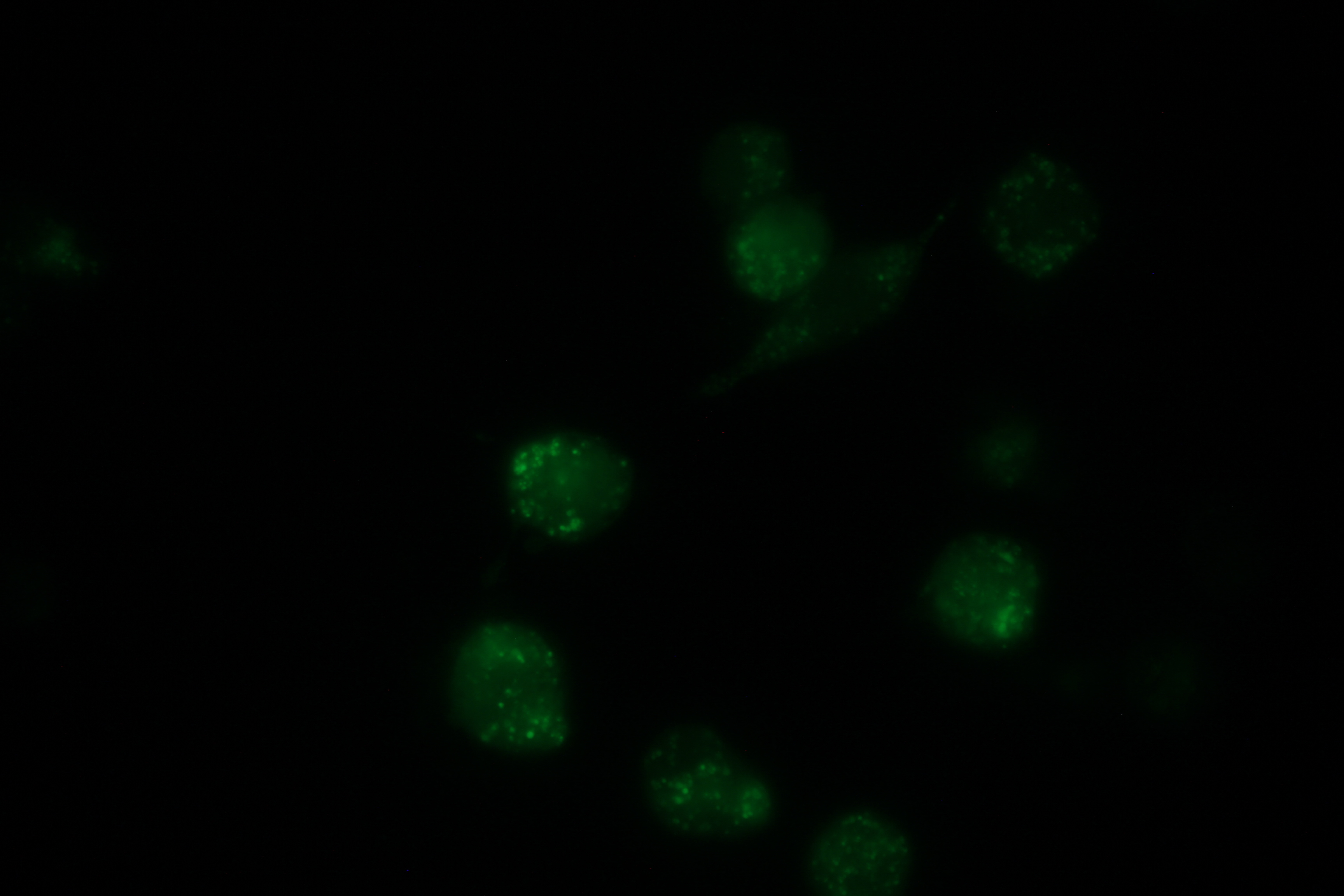

Supplement: Supplementary file 7 — Supplementary Data 4 [file 42003_2021_2408_MOESM7_ESM.zip › Images/Baf GFP, unprocessed, uncropped.jpg]

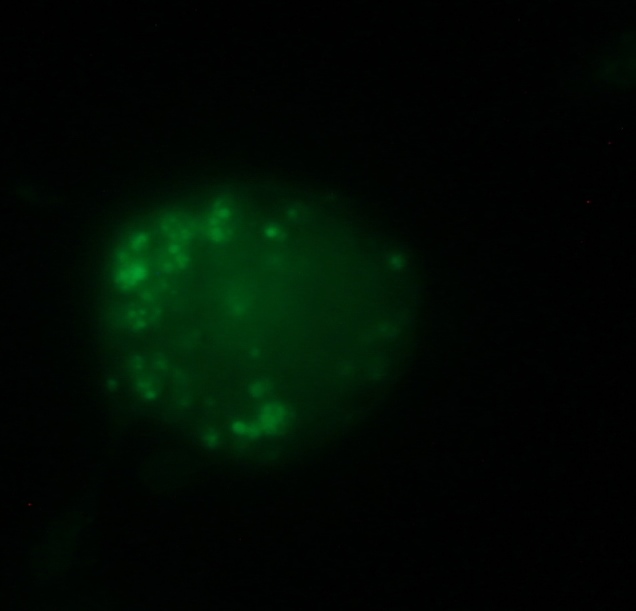

Supplement: Supplementary file 7 — Supplementary Data 4 [file 42003_2021_2408_MOESM7_ESM.zip › Images/Baf GFP.jpg]

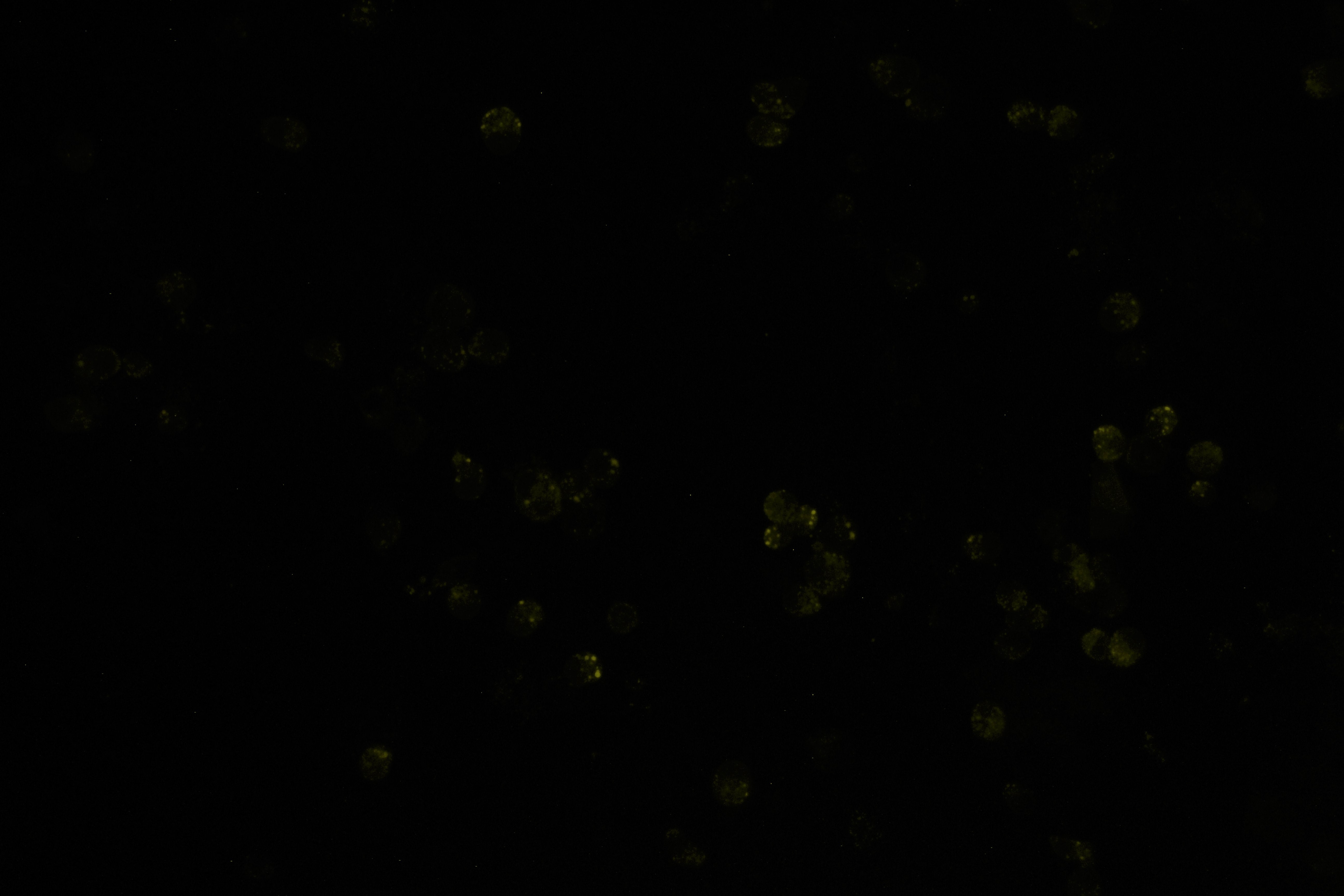

Supplement: Supplementary file 7 — Supplementary Data 4 [file 42003_2021_2408_MOESM7_ESM.zip › Images/Baf merge, low magnification.jpg]

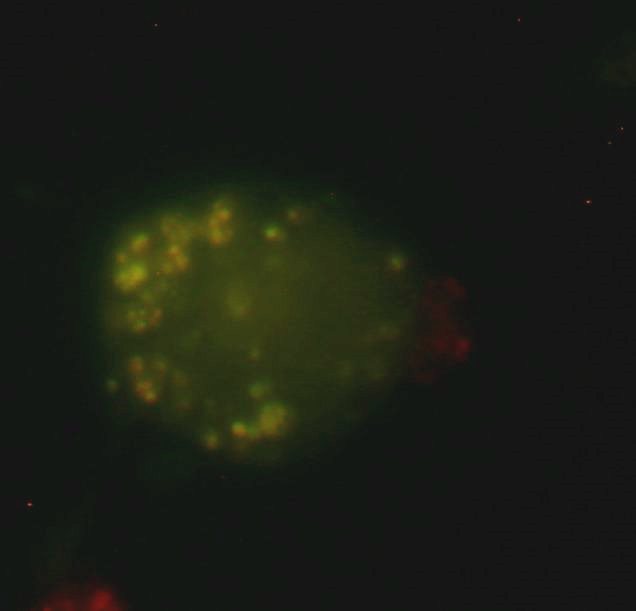

Supplement: Supplementary file 7 — Supplementary Data 4 [file 42003_2021_2408_MOESM7_ESM.zip › Images/BAf Merge.jpg]

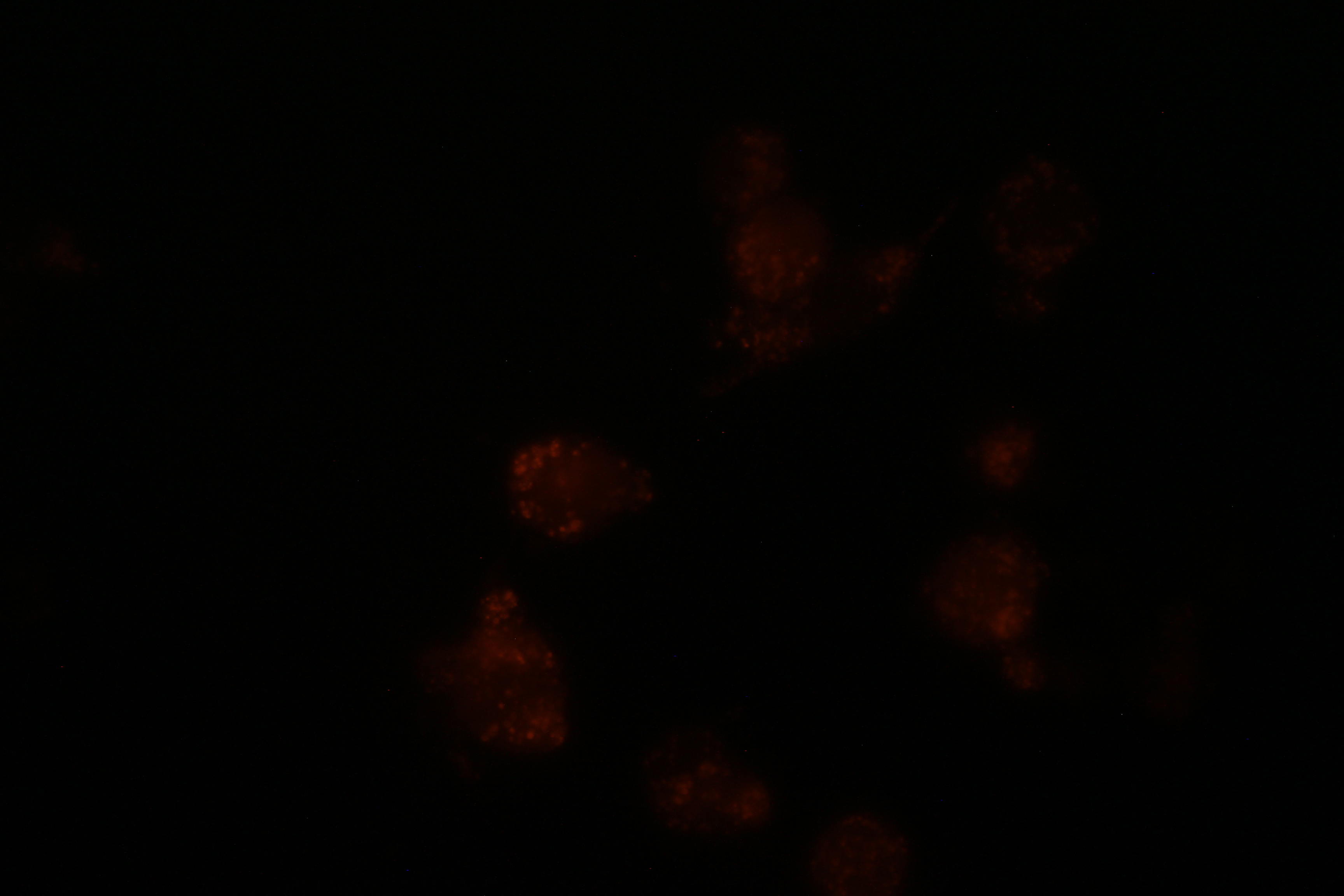

Supplement: Supplementary file 7 — Supplementary Data 4 [file 42003_2021_2408_MOESM7_ESM.zip › Images/Baf RFP unprocessed, uncropped.jpg]

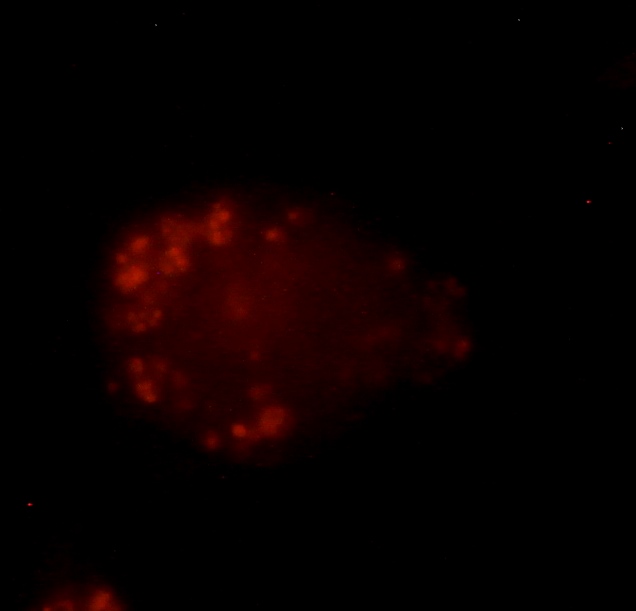

Supplement: Supplementary file 7 — Supplementary Data 4 [file 42003_2021_2408_MOESM7_ESM.zip › Images/Baf RFP.jpg]

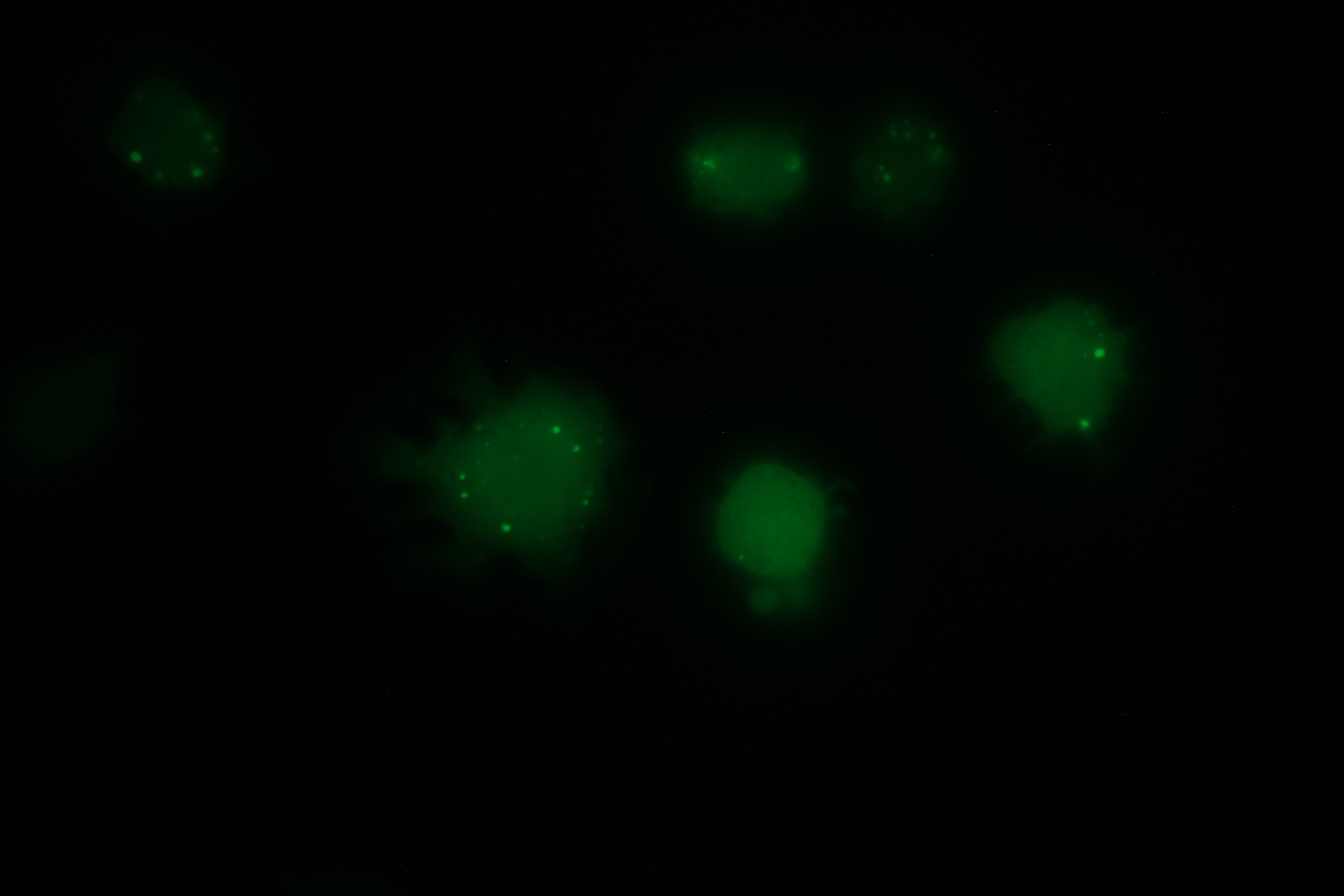

Supplement: Supplementary file 7 — Supplementary Data 4 [file 42003_2021_2408_MOESM7_ESM.zip › Images/LPS GFP unprocessed, uncropped.jpg]

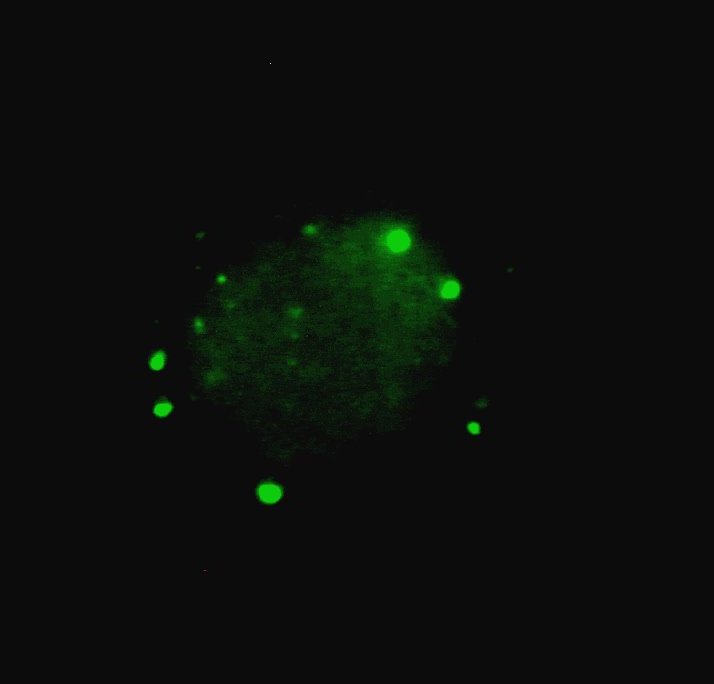

Supplement: Supplementary file 7 — Supplementary Data 4 [file 42003_2021_2408_MOESM7_ESM.zip › Images/LPS GFP.jpg]

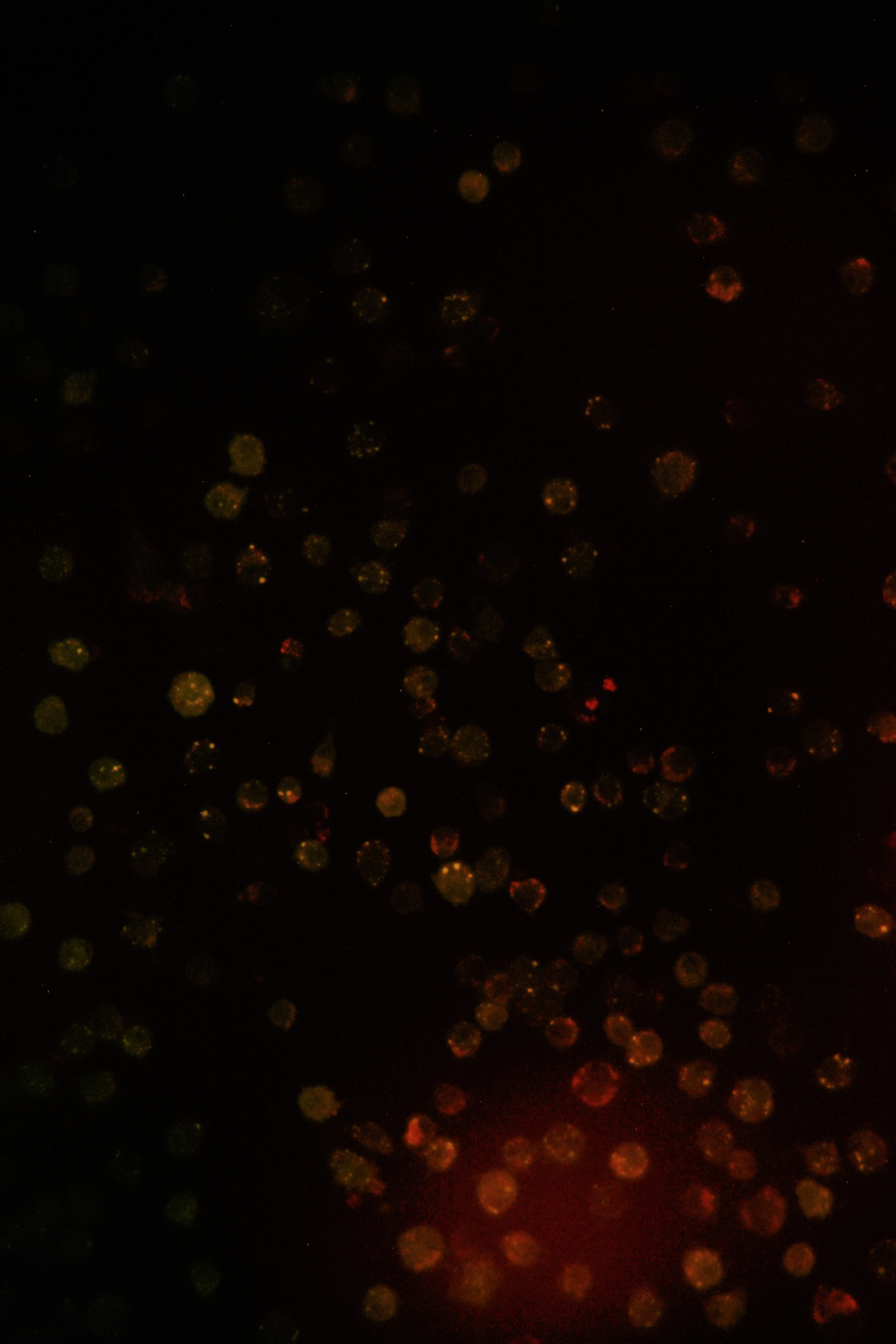

Supplement: Supplementary file 7 — Supplementary Data 4 [file 42003_2021_2408_MOESM7_ESM.zip › Images/LPS merge, low magnification.jpg]

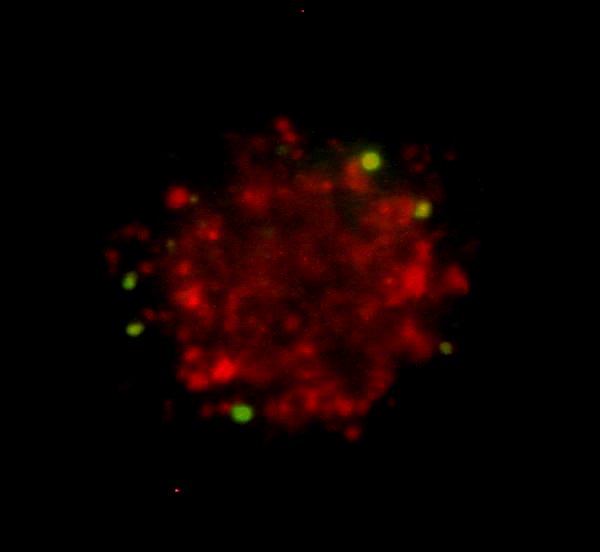

Supplement: Supplementary file 7 — Supplementary Data 4 [file 42003_2021_2408_MOESM7_ESM.zip › Images/LPS Merge.jpg]

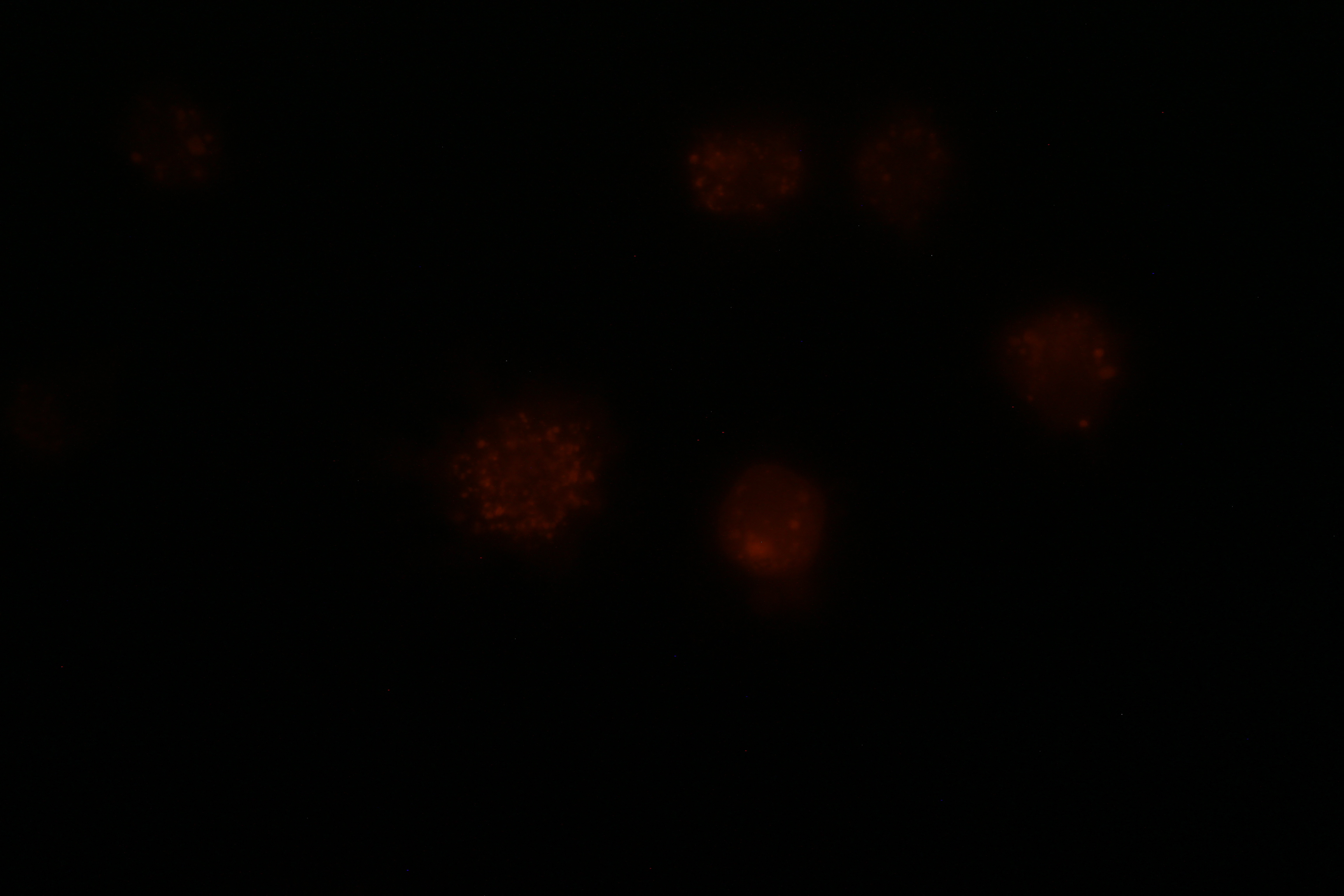

Supplement: Supplementary file 7 — Supplementary Data 4 [file 42003_2021_2408_MOESM7_ESM.zip › Images/LPS RFP unprocessed, uncropped.jpg]

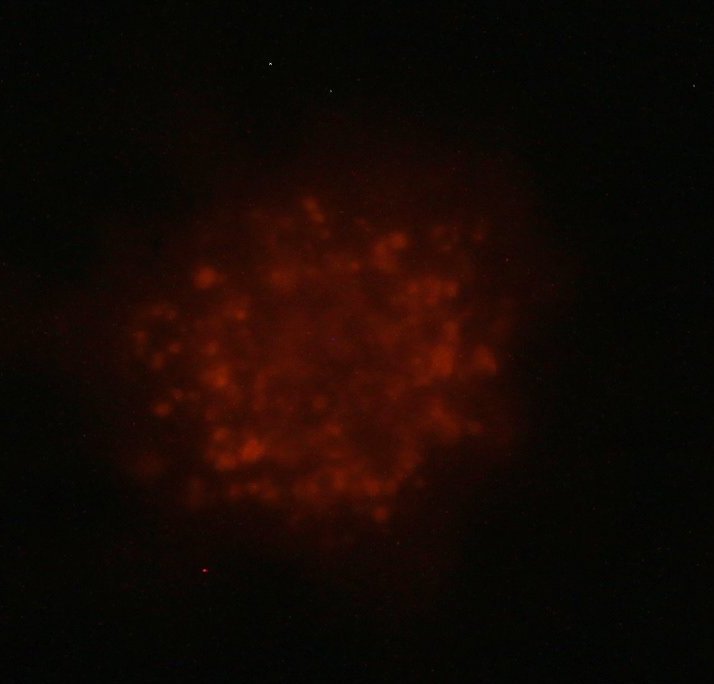

Supplement: Supplementary file 7 — Supplementary Data 4 [file 42003_2021_2408_MOESM7_ESM.zip › Images/LPS RFP.jpg]

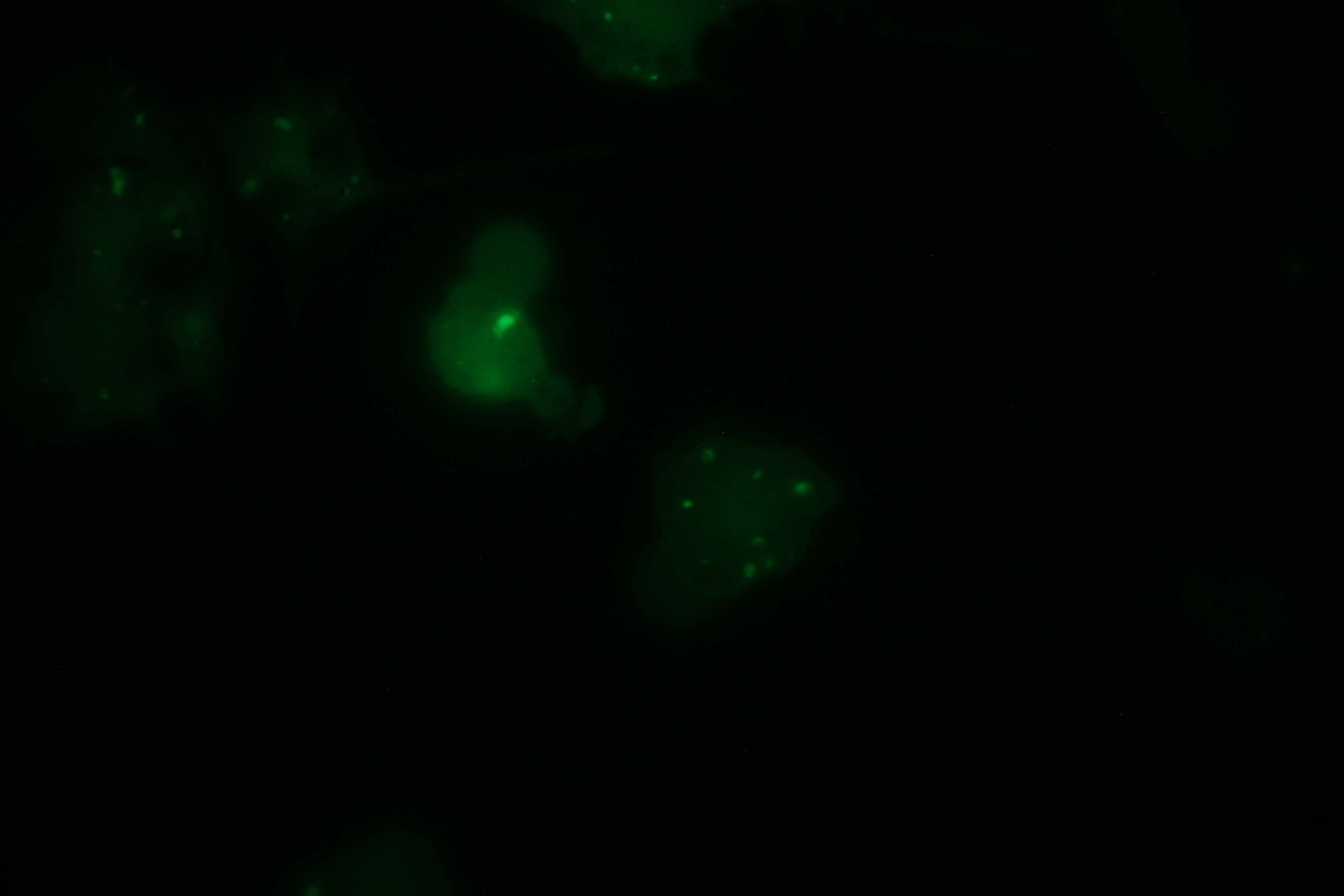

Supplement: Supplementary file 7 — Supplementary Data 4 [file 42003_2021_2408_MOESM7_ESM.zip › Images/PAC GFP unprocessed, uncropped.jpg]

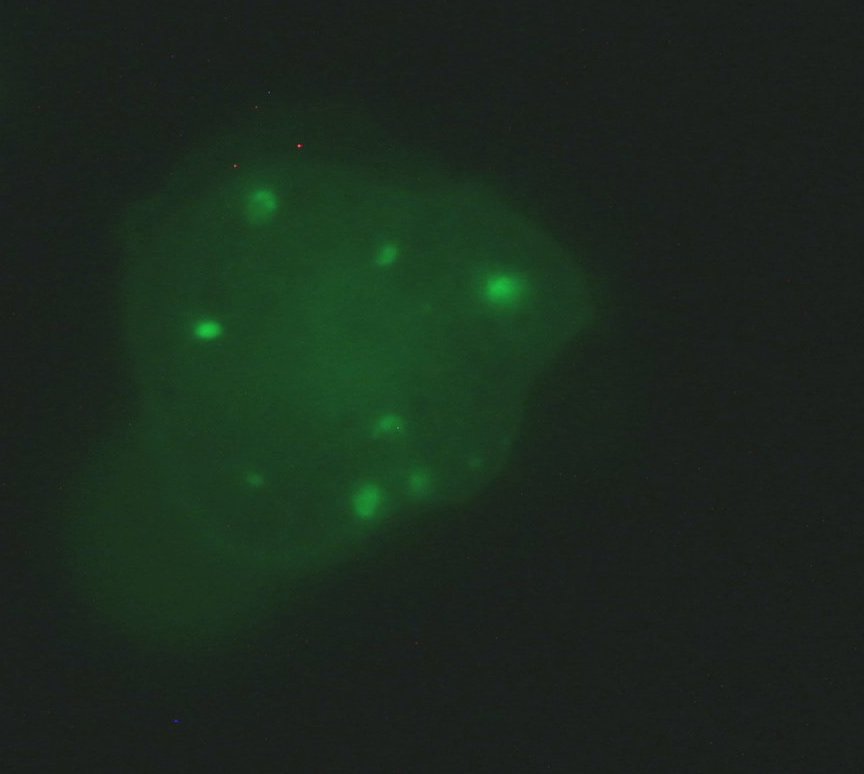

Supplement: Supplementary file 7 — Supplementary Data 4 [file 42003_2021_2408_MOESM7_ESM.zip › Images/PAC GFP.jpg]

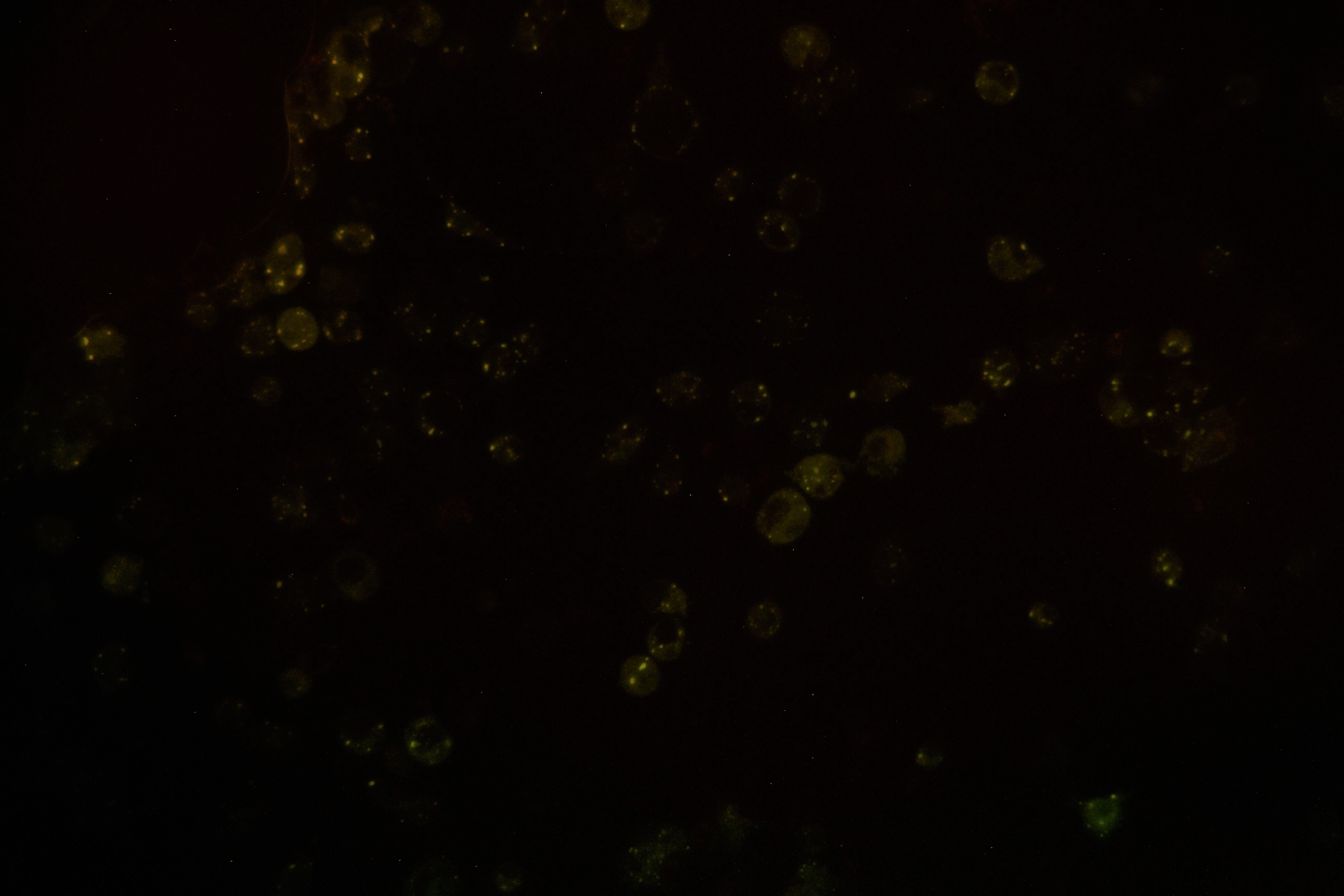

Supplement: Supplementary file 7 — Supplementary Data 4 [file 42003_2021_2408_MOESM7_ESM.zip › Images/PAC merge, low magnification.jpg]

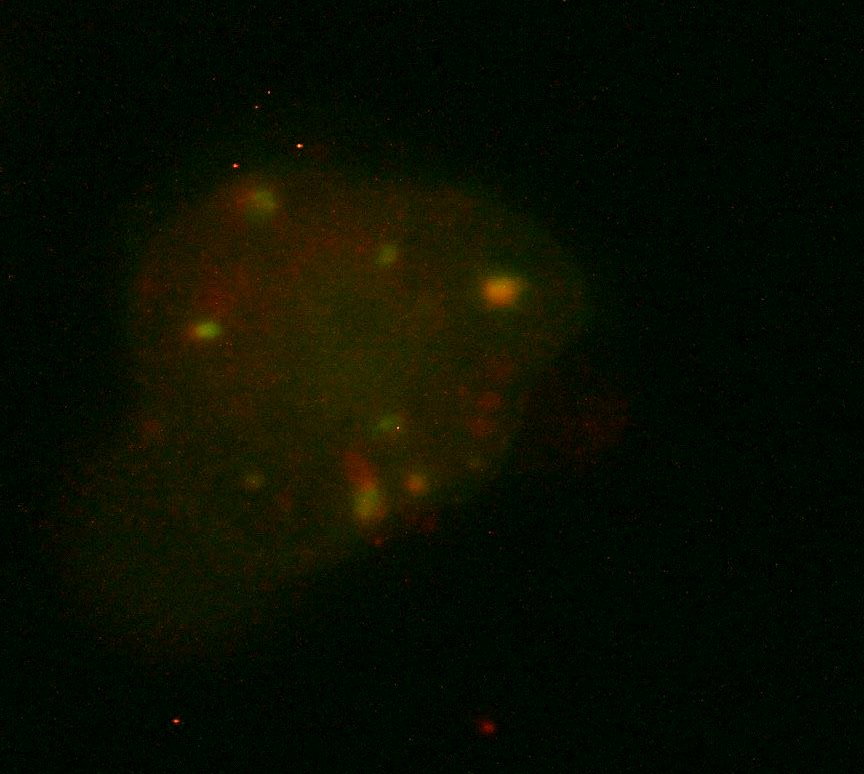

Supplement: Supplementary file 7 — Supplementary Data 4 [file 42003_2021_2408_MOESM7_ESM.zip › Images/PAC Merge.jpg]

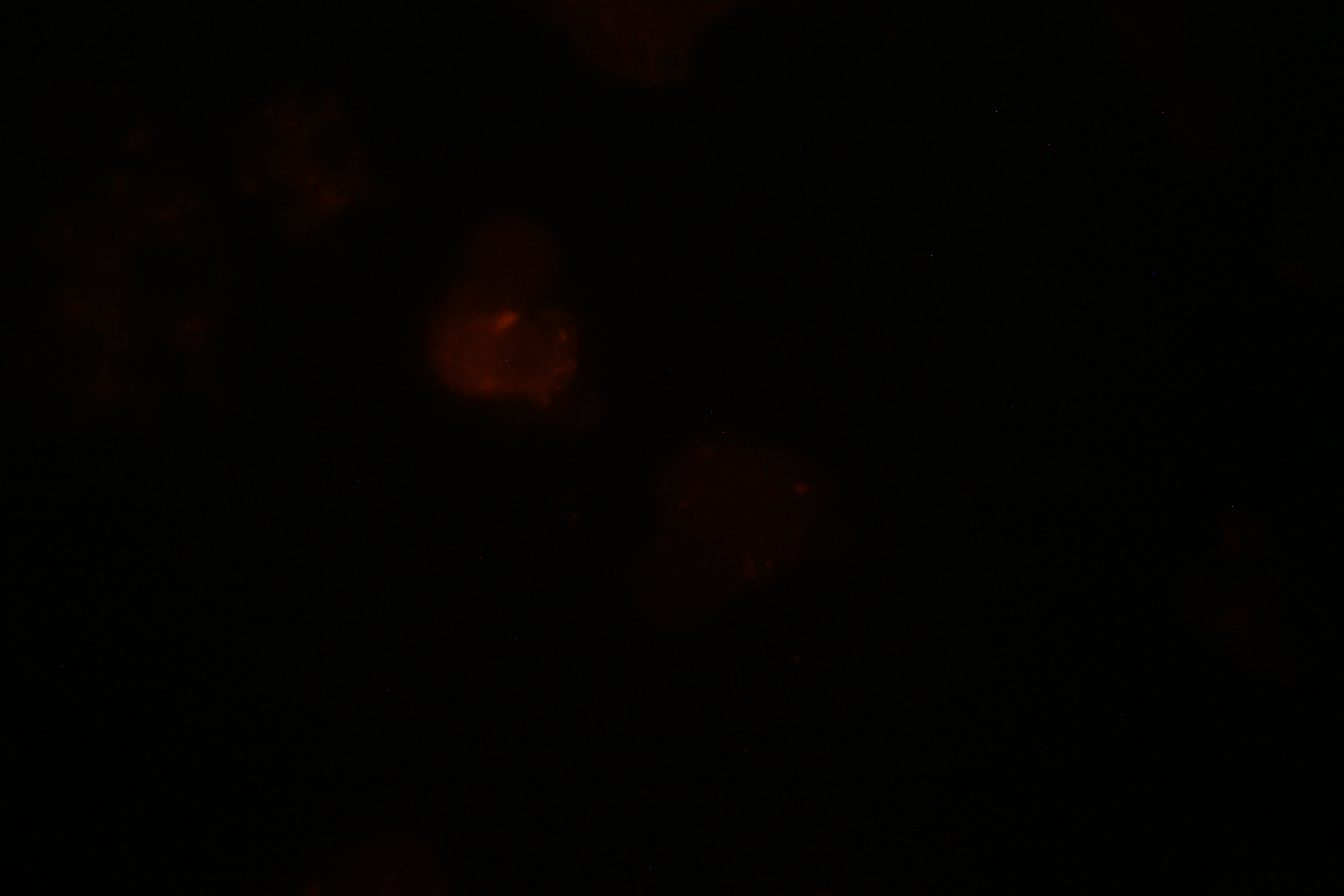

Supplement: Supplementary file 7 — Supplementary Data 4 [file 42003_2021_2408_MOESM7_ESM.zip › Images/PAC RFP unprocessed, uncropped.jpg]

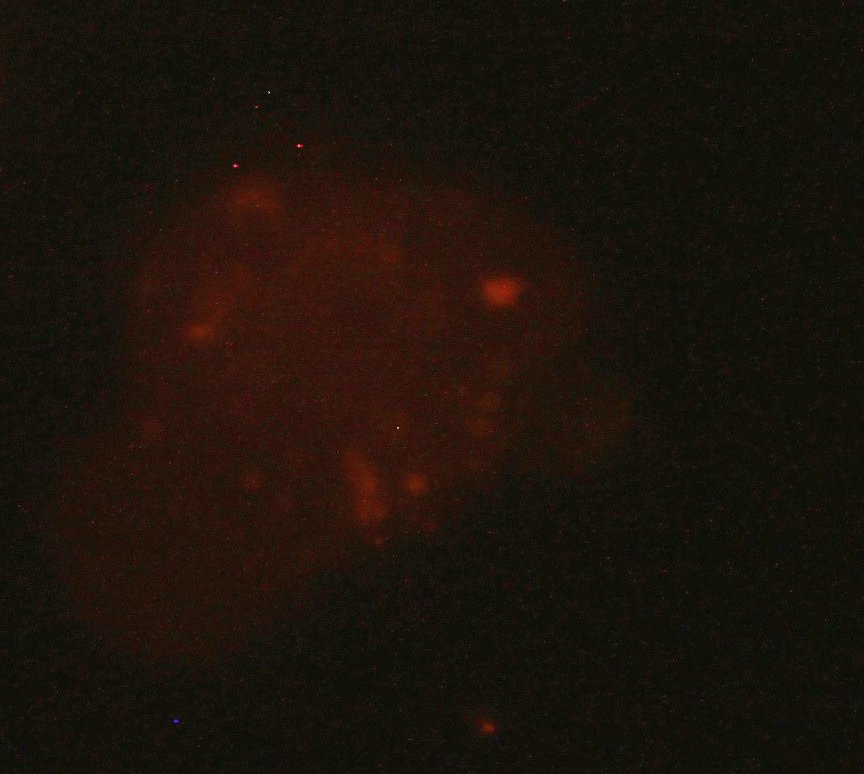

Supplement: Supplementary file 7 — Supplementary Data 4 [file 42003_2021_2408_MOESM7_ESM.zip › Images/PAC RFP.jpg]
