# Supplementary material for: Structure-function analysis of purified proanthocyanidins reveals a role for polymer size in suppressing inflammatory responses
Source: Commun Biol. 2021 Jul 21;4:896. doi: 10.1038/s42003-021-02408-3 (PMC8295316; doi:10.1038/s42003-021-02408-3)
Supplement: Supplementary file 8 — Reporting Summary [file 42003_2021_2408_MOESM8_ESM.pdf]

nature research

Corresponding author(s): Andrew R Williams

Last updated by author(s): Jun 15, 2021

Reporting Summary

Nature Research wishes to improve the reproducibility of the work that we publish. This form provides structure for consistency and transparency in reporting. For further information on Nature Research policies, see our Editorial Policies and the Editorial Policy Checklist.

Statistics

For all statistical analyses, confirm that the following items are present in the figure legend, table legend, main text, or Methods section.

n/a	Confirmed	
☐	☒	The exact sample size (n) for each experimental group/condition, given as a discrete number and unit of measurement
☐	☒	A statement on whether measurements were taken from distinct samples or whether the same sample was measured repeatedly
☐	☒	The statistical test(s) used AND whether they are one- or two-sided<br>*Only common tests should be described solely by name; describe more complex techniques in the Methods section.*
☒	☐	A description of all covariates tested
☐	☒	A description of any assumptions or corrections, such as tests of normality and adjustment for multiple comparisons
☐	☒	A full description of the statistical parameters including central tendency (e.g. means) or other basic estimates (e.g. regression coefficient) AND variation (e.g. standard deviation) or associated estimates of uncertainty (e.g. confidence intervals)
☒	☐	For null hypothesis testing, the test statistic (e.g. F, t, r) with confidence intervals, effect sizes, degrees of freedom and P value noted<br>*Give P values as exact values whenever suitable.*
☒	☐	For Bayesian analysis, information on the choice of priors and Markov chain Monte Carlo settings
☒	☐	For hierarchical and complex designs, identification of the appropriate level for tests and full reporting of outcomes
☒	☐	Estimates of effect sizes (e.g. Cohen's d, Pearson's r), indicating how they were calculated

Our web collection on statistics for biologists contains articles on many of the points above.

Software and code

Policy information about availability of computer code

Data collection	N/A
Data analysis	N/A

For manuscripts utilizing custom algorithms or software that are central to the research but not yet described in published literature, software must be made available to editors and reviewers. We strongly encourage code deposition in a community repository (e.g. GitHub). See the Nature Research guidelines for submitting code & software for further information.

Data

Policy information about availability of data

All manuscripts must include a data availability statement. This statement should provide the following information, where applicable:

- Accession codes, unique identifiers, or web links for publicly available datasets
- A list of figures that have associated raw data
- A description of any restrictions on data availability

Microarray and RNA-seq data is available at GEO under the accession numbers GSE167063 and GSE168138, respectively. All other data is contained within the manuscript and its supplementary files.

Field-specific reporting

Life sciences study design

All studies must disclose on these points even when the disclosure is negative.

Sample size	No statistical tests were used to determine sample sizes. Sample sizes were based on published literature and previous experiments utilizing these techniques in our group.
Data exclusions	No data were excluded from analysis.
Replication	Experimental in vitro data was repeated in independent experiments and successfully confirmed. In vivo studies were not repeated due to the high cost of RNA-sequencing.
Randomization	For in vivo studies, mice were randomly allocated to treatment groups.
Blinding	Blinding was not used during data acquisition and analysis.

Reporting for specific materials, systems and methods

We require information from authors about some types of materials, experimental systems and methods used in many studies. Here, indicate whether each material, system or method listed is relevant to your study. If you are not sure if a list item applies to your research, read the appropriate section before selecting a response.

Materials & experimental systems

n/a	Involved in the study	
☐	☒	Antibodies
☐	☒	Eukaryotic cell lines
☒	☐	Palaeontology and archaeology
☐	☒	Animals and other organisms
☒	☐	Human research participants
☒	☐	Clinical data
☒	☐	Dual use research of concern

Methods

n/a	Involved in the study	
☒	☐	ChIP-seq
☐	☒	Flow cytometry
☒	☐	MRI-based neuroimaging

Antibodies

Antibodies used	Rat anti-mouse TLR4-APC (Clone SA15-21; Cat# 145405, BioLegend)
Validation	Validated by manufacturer.

Eukaryotic cell lines

Policy information about cell lines

Cell line source(s)	ATCC
Authentication	None of the cell lines were authenticated
Mycoplasma contamination	Cell lines were tested negative for Mycoplasma
Commonly misidentified lines (See ICLAC register)	N/A

Animals and other organisms

Policy information about studies involving animals; ARRIVE guidelines recommended for reporting animal research

Laboratory animals	C57BL/6JOlaHsd mice, female, 7 weeks old, obtained from Envigo (Netherlands).
Wild animals	N/A
Field-collected samples	N/A
Ethics oversight	All experiments involving animals were conducted in agreement with the Danish legislation and the Danish Animal Experiments Inspectorate with the license number 2015-15-0201-0076.

Note that full information on the approval of the study protocol must also be provided in the manuscript.

Flow Cytometry

Plots

Confirm that:

- [x] The axis labels state the marker and fluorochrome used (e.g. CD4-FITC).
- [x] The axis scales are clearly visible. Include numbers along axes only for bottom left plot of group (a 'group' is an analysis of identical markers).
- [x] All plots are contour plots with outliers or pseudocolor plots.
- [x] A numerical value for number of cells or percentage (with statistics) is provided.

Methodology

Sample preparation	RAW 264.7 macrophages were harvested using Accutase.
Instrument	Accuri C6
Software	Cflow software (BD Biosciences)
Cell population abundance	no sorting was applied.
Gating strategy	Please see attached supplementary Figure

- [x] Tick this box to confirm that a figure exemplifying the gating strategy is provided in the Supplementary Information.
